# 5-[(4-Benzyl-1*H*-1,2,3-triazol-1-yl)meth­yl]-5*H*-dibenzo[*b*,*f*]azepine

**DOI:** 10.1107/S1600536813018412

**Published:** 2013-07-10

**Authors:** B. C. Manjunath, K. S. Vinay Kumar, S. Madan Kumar, M. P. Sadashiva, N. K. Lokanath

**Affiliations:** a Department of Studies in Physics, Manasagangotri, University of Mysore, Mysore 570 006, India; b Department of Studies in Chemistry, Manasagangotri, University of Mysore, Mysore 570 006, India

## Abstract

In the title compound, C_24_H_20_N_4_, the azepine ring adopts a boat conformation and the dihedral angle between the benzene rings fused to it is 57.95 (8)°. The bond-angle sum at the azepine N atom is 346.6°, indicating a significant deviation from planarity. The triazole ring subtends a dihedral angle of 71.45 (10)° with the terminal phenyl group. A weak intra­molecular C—H⋯N_a_ (a = azepine) inter­action occurs, which closes an *S*(6) ring.

## Related literature

For a related structure and background to isoxazole derivatives, see: Abdoh *et al.* (2013 ▶). 
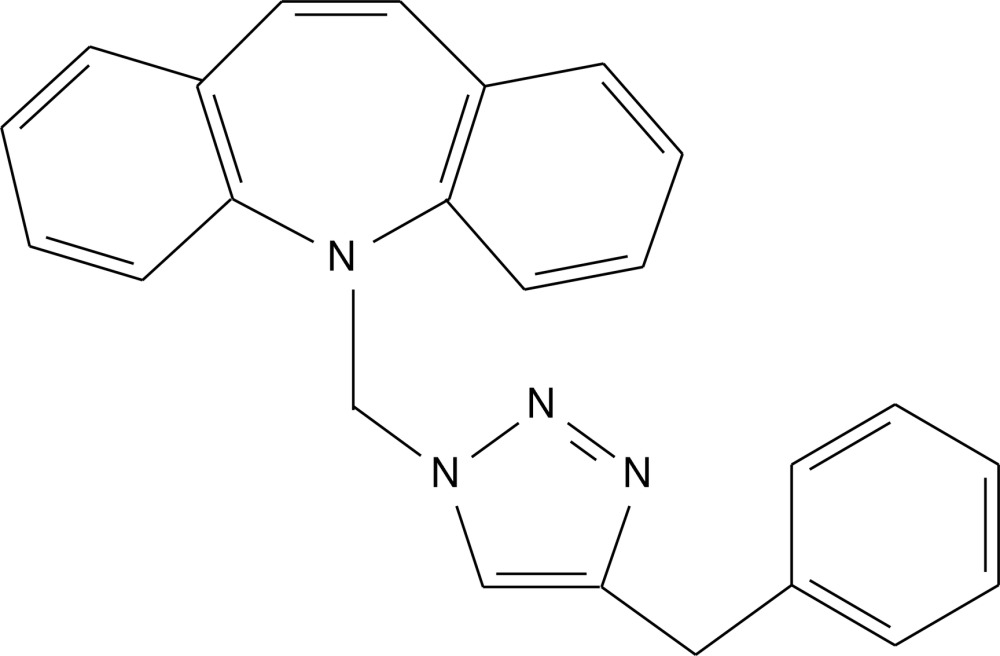


## Experimental

### 

#### Crystal data

C_24_H_20_N_4_
*M* *_r_* = 364.44
Monoclinic, 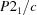

*a* = 9.4394 (10) Å
*b* = 22.206 (3) Å
*c* = 9.4330 (9) Å
β = 107.172 (3)°
*V* = 1889.1 (4) Å^3^
*Z* = 4
Mo *K*α radiation
μ = 0.08 mm^−1^
*T* = 300 K
0.26 × 0.23 × 0.20 mm

#### Data collection

Bruker APEXII CCD diffractometer
22703 measured reflections
3711 independent reflections
2982 reflections with *I* > 2σ(*I*)
*R*_int_ = 0.045

#### Refinement

*R*[*F* ^2^ > 2σ(*F* ^2^)] = 0.045
*wR*(*F* ^2^) = 0.124
*S* = 1.01
3711 reflections
253 parameters
H-atom parameters constrained
Δρ_max_ = 0.16 e Å^−3^
Δρ_min_ = −0.23 e Å^−3^

### 

Data collection: *APEX2* (Bruker, 2005 ▶); cell refinement: *SAINT* (Bruker, 2005 ▶); data reduction: *SAINT*; program(s) used to solve structure: *SHELXS97* (Sheldrick, 2008 ▶); program(s) used to refine structure: *SHELXL97* (Sheldrick, 2008 ▶); molecular graphics: *Mercury* (Macrae *et al.*, 2006 ▶); software used to prepare material for publication: *Mercury*.

## Supplementary Material

Crystal structure: contains datablock(s) global, I. DOI: 10.1107/S1600536813018412/hb7099sup1.cif

Structure factors: contains datablock(s) I. DOI: 10.1107/S1600536813018412/hb7099Isup2.hkl

Supplementary material file. DOI: 10.1107/S1600536813018412/hb7099Isup3.cml

Additional supplementary materials:  crystallographic information; 3D view; checkCIF report

## Figures and Tables

**Table 1:** Hydrogen-bond geometry (Å, °)

*D*—H⋯*A*	*D*—H	H⋯*A*	*D*⋯*A*	*D*—H⋯*A*
C7—H7*A*⋯N4	0.97	2.56	3.173 (2)	121

**Fig. 1.**
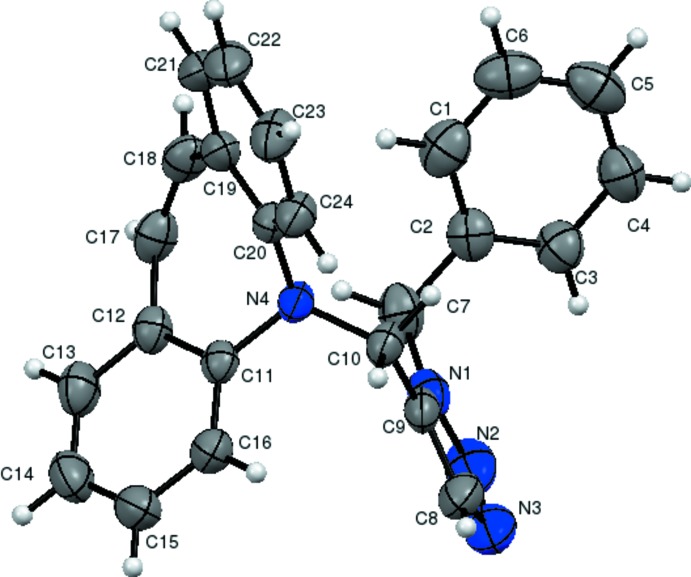
View of the title molecule with 50% probability ellipsoids.

**Fig. 2.**
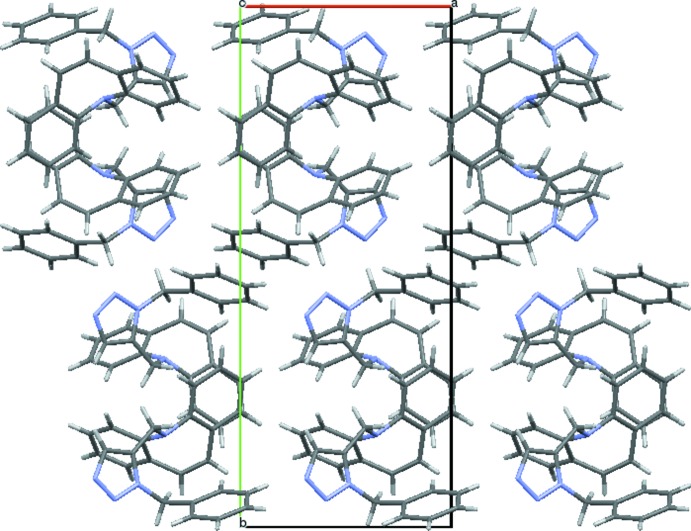
Packing diagram of molecule, viewed along the crystallographic a axis.

## supplementary crystallographic information

### Comment

The title compound was synthesized, crystallized and its crystal structure is presented as part of our investigations on isoxazole derivatives (Abdoh, *et al.*,, 2013)

In the title molecule (Fig. 1.) benzene rings fused to azepine rings are nearly planar and its geometry is similar to 5-(Prop-2–1-yl)-5*H*-dibenzo[*b,f*]azepine: orthorhombic polymorph. Seven-membered azepine ring adopts a boat conformation as indicated by the puckering parameters Q_2_ = 0.7392 (16) Å, Q_3_ = 0.2157 (15) Å, φ_2_ = 178.44 (12) °, φ_3_ = 178.4 (4) °, and the total puckering amplitude, Q_T_ = 0.7700 (15) Å. The title molecule adopts butterfly shape. The dihedral angle between triazole moiety and benzene ring (C1/C2/C3/C4/C5/C6) is 71.45 (10)°. The packing of molecules is shown in the figure 2.

### Experimental

5-(prop-2-yn-1-yl)-5*H*-dibenzo[b,f]azepine (2.1 mmol) was taken in a mixture of dichloromethane and water in the ratio 1:1, cuprous iodide (0.21 mmol) was added followed by sodium ascorbate (0.21 mmol) at room temperature. After 10 minutes, benzyl azide was added (2.3 mmol) at room temperature. Then, the resulting reaction mixture was allowed for stirring upto 6 h. After completion of reaction (monitored by TLC), the reaction mixture was diluted with water (50 ml). The aqueous layer was extracted with ethyl acetate (3 *x* 20 ml), the combined ethyl acetate layer was washed with brine solution (2 *x* 25 ml). Then, the organic layer was dried over anhydrous sodium sulfate, filtered and concentrated under reduced pressure to afford crude, which was purified by column chromatography over silica gel (60–120 mesh) using Hexane: Ethyl acetate mixture in 8:2 ratios as eluent. The pure compound was crystallized in ethyl acetate and hexane to obtain light red blocks.

^1^H NMR (DMSO-d6, 300 MHz): δ 7.33 (t, J=7.2 Hz, 2H), 7.13 (t, J=8.7 Hz, 4H), 7.02 (t, J=7.2 Hz, 2H), 6.75 (s, 2H), 4.51 (d, J=1.8 Hz, 2H), 3.08 (s, 1H).

MS (*M*^+^+1): 232. Melting point (°C): 90 (Uncorrected)

### Refinement

All the hydrogen atoms of the compound are fixed geometrically (C—H= 0.93–0.97 Å) and allowed to ride on their parent atoms.

### Crystal data

**Table:**

C_24_H_20_N_4_	*F*(000) = 768
*M**_r_* = 364.44	*D*_x_ = 1.281 Mg m^−^^3^
Monoclinic, *P*2_1_/*c*	Mo *K*α radiation, λ = 0.71073 Å
Hall symbol: -P 2ybc	Cell parameters from 3711 reflections
*a* = 9.4394 (10) Å	θ = 1.8–26.0°
*b* = 22.206 (3) Å	µ = 0.08 mm^−^^1^
*c* = 9.4330 (9) Å	*T* = 300 K
β = 107.172 (3)°	Block, red
*V* = 1889.1 (4) Å^3^	0.26 × 0.23 × 0.20 mm
*Z* = 4	

### Data collection

**Table:**

Bruker APEXII CCD diffractometer	*R*_int_ = 0.045
Radiation source: graphite	θ_max_ = 26.0°, θ_min_ = 1.8°
φ and ω scans	*h* = −11→11
22703 measured reflections	*k* = −27→26
3711 independent reflections	*l* = −11→11
2982 reflections with *I* > 2σ(*I*)	

### Refinement

**Table:**

Refinement on *F*^2^	Primary atom site location: structure-invariant direct methods
Least-squares matrix: full	Secondary atom site location: difference Fourier map
*R*[*F*^2^ > 2σ(*F*^2^)] = 0.045	Hydrogen site location: inferred from neighbouring sites
*wR*(*F*^2^) = 0.124	H-atom parameters constrained
*S* = 1.01	*w* = 1/[σ^2^(*F*_o_^2^) + (0.0634*P*)^2^ + 0.445*P*] where *P* = (*F*_o_^2^ + 2*F*_c_^2^)/3
3711 reflections	(Δ/σ)_max_ < 0.001
253 parameters	Δρ_max_ = 0.16 e Å^−^^3^
0 restraints	Δρ_min_ = −0.23 e Å^−^^3^

### Special details

**Table:**

Geometry. Bond distances, angles *etc*. have been calculated using the rounded fractional coordinates. All su's are estimated from the variances of the (full) variance-covariance matrix. The cell e.s.d.'s are taken into account in the estimation of distances, angles and torsion angles
Refinement. Refinement on *F*^2^ for ALL reflections except those flagged by the user for potential systematic errors. Weighted *R*-factors *wR* and all goodnesses of fit *S* are based on *F*^2^, conventional *R*-factors *R* are based on *F*, with *F* set to zero for negative *F*^2^. The observed criterion of *F*^2^ > σ(*F*^2^) is used only for calculating -*R*-factor-obs *etc*. and is not relevant to the choice of reflections for refinement. *R*-factors based on *F*^2^ are statistically about twice as large as those based on *F*, and *R*-factors based on ALL data will be even larger.

### Fractional atomic coordinates and isotropic or equivalent isotropic displacement parameters (Å^2^)

**Table:**

	*x*	*y*	*z*	*U*_iso_*/*U*_eq_	
N1	0.48315 (15)	0.41800 (6)	0.71285 (15)	0.0485 (4)	
N2	0.59240 (19)	0.44728 (7)	0.81571 (19)	0.0671 (6)	
N3	0.68625 (18)	0.40672 (9)	0.89031 (18)	0.0687 (6)	
N4	0.35717 (13)	0.32303 (5)	0.47460 (12)	0.0358 (3)	
C1	0.0910 (2)	0.43114 (8)	0.5802 (2)	0.0602 (6)	
C2	0.22436 (19)	0.45366 (6)	0.66850 (18)	0.0475 (5)	
C3	0.2290 (2)	0.48013 (7)	0.8030 (2)	0.0536 (6)	
C4	0.1028 (2)	0.48466 (8)	0.8465 (2)	0.0605 (6)	
C5	−0.0294 (2)	0.46273 (8)	0.7577 (3)	0.0667 (7)	
C6	−0.0358 (2)	0.43573 (8)	0.6250 (3)	0.0690 (7)	
C7	0.3606 (2)	0.45281 (7)	0.6163 (2)	0.0562 (6)	
C8	0.63739 (19)	0.35255 (9)	0.83410 (18)	0.0551 (5)	
C9	0.50799 (17)	0.35803 (7)	0.72201 (15)	0.0410 (4)	
C10	0.40884 (17)	0.31033 (7)	0.63385 (15)	0.0408 (4)	
C11	0.46560 (15)	0.33324 (6)	0.39878 (15)	0.0357 (4)	
C12	0.42678 (17)	0.37101 (6)	0.27384 (16)	0.0404 (4)	
C13	0.5336 (2)	0.38298 (7)	0.20193 (19)	0.0527 (6)	
C14	0.6730 (2)	0.35838 (9)	0.2500 (2)	0.0600 (6)	
C15	0.71005 (19)	0.32105 (8)	0.3721 (2)	0.0553 (6)	
C16	0.60713 (17)	0.30859 (7)	0.44645 (17)	0.0445 (5)	
C17	0.27898 (19)	0.39760 (7)	0.21582 (17)	0.0478 (5)	
C18	0.15052 (19)	0.37247 (7)	0.21404 (18)	0.0484 (5)	
C19	0.13042 (16)	0.31254 (7)	0.26945 (16)	0.0402 (4)	
C20	0.23460 (15)	0.28684 (6)	0.39198 (14)	0.0353 (4)	
C21	0.00604 (17)	0.27797 (8)	0.19635 (18)	0.0523 (5)	
C22	−0.01154 (19)	0.21998 (9)	0.2393 (2)	0.0582 (6)	
C23	0.0936 (2)	0.19481 (8)	0.35653 (19)	0.0544 (6)	
C24	0.21656 (18)	0.22797 (7)	0.43327 (17)	0.0447 (5)	
H1	0.08630	0.41280	0.49030	0.0720*	
H3	0.31830	0.49490	0.86420	0.0640*	
H4	0.10710	0.50270	0.93670	0.0730*	
H5	−0.11480	0.46610	0.78720	0.0800*	
H6	−0.12530	0.42050	0.56520	0.0830*	
H7A	0.33470	0.43590	0.51710	0.0670*	
H7B	0.39350	0.49390	0.61070	0.0670*	
H8	0.68500	0.31630	0.86690	0.0660*	
H10A	0.46180	0.27230	0.64940	0.0490*	
H10B	0.32350	0.30600	0.67030	0.0490*	
H13	0.50970	0.40820	0.11960	0.0630*	
H14	0.74230	0.36690	0.20030	0.0720*	
H15	0.80430	0.30420	0.40460	0.0660*	
H16	0.63290	0.28350	0.52900	0.0530*	
H17	0.27440	0.43610	0.17600	0.0570*	
H18	0.06540	0.39520	0.17370	0.0580*	
H21	−0.06640	0.29470	0.11680	0.0630*	
H22	−0.09480	0.19790	0.18860	0.0700*	
H23	0.08250	0.15540	0.38470	0.0650*	
H24	0.28760	0.21070	0.51300	0.0540*	

### Atomic displacement parameters (Å^2^)

**Table:**

	*U*^11^	*U*^22^	*U*^33^	*U*^12^	*U*^13^	*U*^23^
N1	0.0535 (8)	0.0486 (7)	0.0494 (8)	−0.0117 (6)	0.0243 (7)	−0.0132 (6)
N2	0.0721 (11)	0.0676 (10)	0.0667 (10)	−0.0319 (8)	0.0284 (9)	−0.0278 (8)
N3	0.0584 (9)	0.0904 (12)	0.0560 (9)	−0.0262 (9)	0.0148 (8)	−0.0203 (9)
N4	0.0388 (6)	0.0403 (6)	0.0274 (6)	−0.0056 (5)	0.0084 (5)	−0.0010 (5)
C1	0.0747 (12)	0.0467 (9)	0.0548 (10)	0.0002 (8)	0.0122 (9)	−0.0074 (8)
C2	0.0605 (10)	0.0334 (7)	0.0510 (9)	0.0017 (7)	0.0203 (8)	−0.0009 (6)
C3	0.0587 (10)	0.0483 (9)	0.0551 (10)	−0.0030 (7)	0.0190 (8)	−0.0087 (7)
C4	0.0681 (12)	0.0592 (10)	0.0615 (11)	0.0058 (9)	0.0305 (10)	−0.0041 (8)
C5	0.0601 (12)	0.0564 (11)	0.0906 (15)	0.0077 (9)	0.0329 (11)	0.0111 (10)
C6	0.0547 (11)	0.0542 (10)	0.0881 (15)	−0.0045 (8)	0.0056 (10)	0.0011 (10)
C7	0.0755 (12)	0.0450 (9)	0.0562 (10)	0.0010 (8)	0.0319 (9)	−0.0008 (7)
C8	0.0511 (9)	0.0718 (11)	0.0393 (8)	−0.0110 (8)	0.0085 (7)	−0.0070 (8)
C9	0.0454 (8)	0.0498 (8)	0.0310 (7)	−0.0091 (6)	0.0164 (6)	−0.0051 (6)
C10	0.0476 (8)	0.0450 (8)	0.0285 (7)	−0.0077 (6)	0.0092 (6)	−0.0005 (6)
C11	0.0412 (7)	0.0350 (7)	0.0309 (7)	−0.0060 (5)	0.0108 (6)	−0.0053 (5)
C12	0.0506 (8)	0.0365 (7)	0.0350 (7)	−0.0059 (6)	0.0139 (7)	−0.0016 (6)
C13	0.0663 (11)	0.0538 (9)	0.0420 (9)	−0.0098 (8)	0.0222 (8)	0.0044 (7)
C14	0.0581 (11)	0.0758 (12)	0.0545 (10)	−0.0136 (9)	0.0297 (9)	−0.0049 (9)
C15	0.0427 (9)	0.0717 (11)	0.0528 (10)	−0.0017 (8)	0.0159 (8)	−0.0090 (8)
C16	0.0449 (8)	0.0506 (8)	0.0368 (8)	−0.0016 (7)	0.0101 (7)	−0.0024 (6)
C17	0.0645 (10)	0.0375 (8)	0.0393 (8)	0.0062 (7)	0.0123 (7)	0.0068 (6)
C18	0.0516 (9)	0.0478 (8)	0.0419 (8)	0.0150 (7)	0.0079 (7)	0.0031 (6)
C19	0.0362 (7)	0.0493 (8)	0.0352 (7)	0.0042 (6)	0.0106 (6)	−0.0033 (6)
C20	0.0364 (7)	0.0410 (7)	0.0293 (7)	−0.0022 (6)	0.0109 (6)	−0.0046 (5)
C21	0.0367 (8)	0.0746 (11)	0.0409 (8)	0.0023 (7)	0.0041 (7)	−0.0035 (8)
C22	0.0477 (9)	0.0767 (12)	0.0487 (10)	−0.0226 (8)	0.0118 (8)	−0.0122 (8)
C23	0.0641 (11)	0.0534 (9)	0.0448 (9)	−0.0206 (8)	0.0145 (8)	−0.0045 (7)
C24	0.0500 (9)	0.0468 (8)	0.0351 (8)	−0.0079 (7)	0.0090 (7)	0.0003 (6)

### Geometric parameters (Å, º)

**Table:**

N1—N2	1.355 (2)	C19—C20	1.399 (2)
N1—C7	1.464 (2)	C19—C21	1.402 (2)
N1—C9	1.351 (2)	C20—C24	1.389 (2)
N2—N3	1.313 (3)	C21—C22	1.375 (3)
N3—C8	1.340 (3)	C22—C23	1.368 (3)
N4—C10	1.4634 (17)	C23—C24	1.385 (2)
N4—C11	1.4293 (19)	C1—H1	0.9300
N4—C20	1.4351 (18)	C3—H3	0.9300
C1—C2	1.382 (3)	C4—H4	0.9300
C1—C6	1.386 (3)	C5—H5	0.9300
C2—C3	1.387 (2)	C6—H6	0.9300
C2—C7	1.507 (3)	C7—H7A	0.9700
C3—C4	1.373 (3)	C7—H7B	0.9700
C4—C5	1.371 (3)	C8—H8	0.9300
C5—C6	1.373 (4)	C10—H10A	0.9700
C8—C9	1.365 (2)	C10—H10B	0.9700
C9—C10	1.493 (2)	C13—H13	0.9300
C11—C12	1.404 (2)	C14—H14	0.9300
C11—C16	1.390 (2)	C15—H15	0.9300
C12—C13	1.397 (2)	C16—H16	0.9300
C12—C17	1.464 (2)	C17—H17	0.9300
C13—C14	1.372 (3)	C18—H18	0.9300
C14—C15	1.378 (3)	C21—H21	0.9300
C15—C16	1.384 (2)	C22—H22	0.9300
C17—C18	1.330 (3)	C23—H23	0.9300
C18—C19	1.463 (2)	C24—H24	0.9300
			
N2—N1—C7	119.12 (13)	C20—C24—C23	120.48 (15)
N2—N1—C9	110.26 (13)	C2—C1—H1	120.00
C7—N1—C9	130.61 (14)	C6—C1—H1	120.00
N1—N2—N3	107.76 (15)	C2—C3—H3	120.00
N2—N3—C8	107.75 (16)	C4—C3—H3	120.00
C10—N4—C11	118.25 (12)	C3—C4—H4	120.00
C10—N4—C20	114.87 (11)	C5—C4—H4	120.00
C11—N4—C20	113.50 (11)	C4—C5—H5	120.00
C2—C1—C6	120.31 (18)	C6—C5—H5	120.00
C1—C2—C3	118.80 (17)	C1—C6—H6	120.00
C1—C2—C7	120.80 (15)	C5—C6—H6	120.00
C3—C2—C7	120.31 (16)	N1—C7—H7A	109.00
C2—C3—C4	120.55 (17)	N1—C7—H7B	109.00
C3—C4—C5	120.32 (18)	C2—C7—H7A	109.00
C4—C5—C6	119.96 (19)	C2—C7—H7B	109.00
C1—C6—C5	120.0 (2)	H7A—C7—H7B	108.00
N1—C7—C2	113.40 (14)	N3—C8—H8	125.00
N3—C8—C9	110.56 (17)	C9—C8—H8	125.00
N1—C9—C8	103.67 (14)	N4—C10—H10A	109.00
N1—C9—C10	126.54 (14)	N4—C10—H10B	109.00
C8—C9—C10	129.70 (15)	C9—C10—H10A	109.00
N4—C10—C9	113.52 (12)	C9—C10—H10B	109.00
N4—C11—C12	117.83 (13)	H10A—C10—H10B	108.00
N4—C11—C16	122.52 (12)	C12—C13—H13	119.00
C12—C11—C16	119.64 (14)	C14—C13—H13	119.00
C11—C12—C13	118.32 (15)	C13—C14—H14	120.00
C11—C12—C17	122.57 (14)	C15—C14—H14	120.00
C13—C12—C17	119.11 (14)	C14—C15—H15	120.00
C12—C13—C14	121.53 (16)	C16—C15—H15	120.00
C13—C14—C15	119.85 (18)	C11—C16—H16	120.00
C14—C15—C16	120.07 (17)	C15—C16—H16	120.00
C11—C16—C15	120.59 (15)	C12—C17—H17	117.00
C12—C17—C18	126.86 (14)	C18—C17—H17	117.00
C17—C18—C19	126.24 (16)	C17—C18—H18	117.00
C18—C19—C20	122.19 (14)	C19—C18—H18	117.00
C18—C19—C21	120.05 (14)	C19—C21—H21	119.00
C20—C19—C21	117.73 (14)	C22—C21—H21	119.00
N4—C20—C19	118.53 (12)	C21—C22—H22	120.00
N4—C20—C24	121.38 (12)	C23—C22—H22	120.00
C19—C20—C24	120.08 (14)	C22—C23—H23	120.00
C19—C21—C22	121.63 (16)	C24—C23—H23	120.00
C21—C22—C23	119.93 (17)	C20—C24—H24	120.00
C22—C23—C24	120.09 (17)	C23—C24—H24	120.00
			
C7—N1—N2—N3	178.33 (15)	N3—C8—C9—C10	−175.78 (16)
C9—N1—N2—N3	−0.1 (2)	N1—C9—C10—N4	48.1 (2)
N2—N1—C7—C2	−97.83 (18)	C8—C9—C10—N4	−136.11 (17)
C9—N1—C7—C2	80.2 (2)	N4—C11—C12—C13	177.82 (13)
N2—N1—C9—C8	−0.40 (18)	N4—C11—C12—C17	−2.8 (2)
N2—N1—C9—C10	176.29 (15)	C16—C11—C12—C13	−0.6 (2)
C7—N1—C9—C8	−178.59 (17)	C16—C11—C12—C17	178.82 (14)
C7—N1—C9—C10	−1.9 (3)	N4—C11—C16—C15	−178.14 (14)
N1—N2—N3—C8	0.6 (2)	C12—C11—C16—C15	0.1 (2)
N2—N3—C8—C9	−0.9 (2)	C11—C12—C13—C14	0.6 (2)
C11—N4—C10—C9	56.44 (17)	C17—C12—C13—C14	−178.83 (16)
C20—N4—C10—C9	−165.14 (13)	C11—C12—C17—C18	−35.1 (2)
C10—N4—C11—C12	−150.96 (13)	C13—C12—C17—C18	144.31 (17)
C10—N4—C11—C16	27.36 (19)	C12—C13—C14—C15	−0.2 (3)
C20—N4—C11—C12	70.09 (15)	C13—C14—C15—C16	−0.3 (3)
C20—N4—C11—C16	−111.60 (15)	C14—C15—C16—C11	0.3 (3)
C10—N4—C20—C19	146.64 (13)	C12—C17—C18—C19	−0.3 (3)
C10—N4—C20—C24	−31.99 (19)	C17—C18—C19—C20	33.4 (2)
C11—N4—C20—C19	−72.97 (16)	C17—C18—C19—C21	−144.41 (17)
C11—N4—C20—C24	108.41 (15)	C18—C19—C20—N4	6.5 (2)
C6—C1—C2—C3	0.8 (2)	C18—C19—C20—C24	−174.85 (15)
C6—C1—C2—C7	−175.74 (16)	C21—C19—C20—N4	−175.64 (13)
C2—C1—C6—C5	0.0 (3)	C21—C19—C20—C24	3.0 (2)
C1—C2—C3—C4	−1.0 (2)	C18—C19—C21—C22	175.63 (16)
C7—C2—C3—C4	175.58 (15)	C20—C19—C21—C22	−2.3 (2)
C1—C2—C7—N1	−119.86 (17)	N4—C20—C24—C23	176.75 (15)
C3—C2—C7—N1	63.67 (18)	C19—C20—C24—C23	−1.9 (2)
C2—C3—C4—C5	0.4 (3)	C19—C21—C22—C23	0.3 (3)
C3—C4—C5—C6	0.4 (3)	C21—C22—C23—C24	0.9 (3)
C4—C5—C6—C1	−0.6 (3)	C22—C23—C24—C20	−0.2 (3)
N3—C8—C9—N1	0.77 (19)		

### Hydrogen-bond geometry (Å, º)

**Table:**

*D*—H···*A*	*D*—H	H···*A*	*D*···*A*	*D*—H···*A*
C7—H7*A*···N4	0.97	2.56	3.173 (2)	121
